# Thermochemical electronegativities of the elements

**DOI:** 10.1038/s41467-021-22429-0

**Published:** 2021-04-07

**Authors:** Christian Tantardini, Artem R. Oganov

**Affiliations:** 1 Skolkovo Institute of Science and Technology, Bolshoi Boulevard 30, Moscow, 121025 Russian Federation; 2 Institute of Solid State Chemistry and Mechanochemistry SB RAS, 630128, Kutateladze 18, Novosibirsk, Russian Federation

**Keywords:** History of chemistry, Chemical bonding, Method development

## Abstract

Electronegativity is a key property of the elements. Being useful in rationalizing stability, structure and properties of molecules and solids, it has shaped much of the thinking in the fields of structural chemistry and solid state chemistry and physics. There are many definitions of electronegativity, which can be roughly classified as either spectroscopic (these are defined for isolated atoms) or thermochemical (characterizing bond energies and heats of formation of compounds). The most widely used is the thermochemical Pauling’s scale, where electronegativities have units of eV^1/2^. Here we identify drawbacks in the definition of Pauling’s electronegativity scale—and, correcting them, arrive at our thermochemical scale, where electronegativities are dimensionless numbers. Our scale displays intuitively correct trends for the 118 elements and leads to an improved description of chemical bonding (e.g., bond polarity) and thermochemistry.

## Abstract (web summary)

Pauling’s electronegativity scale has a fundamental value and uses accessible thermochemical data, but fails at predicting the bonding behavior for several elements. The authors propose their thermochemical scale based on experimental dissociation energies that provides dimensionless values for the electronegativity and recovers the correct trends throughout the periodic table.

## Introduction

Electronegativity is defined as the tendency of an atom to attract electron density, i.e., to polarize the chemical bond. The concept of electronegativity can be traced back to 1819 when great Jöns Jacob Berzelius divided the elements into electropositive and electronegative^1^. This was already useful, even as a qualitative concept that arrived well before the discovery of the electron in 1897^2^. Then, in 1916, Gilbert Newton Lewis formulated his theory of chemical bonding, according to which chemical bond is a result of sharing valence electrons.^3^ Development of this theory has led Linus Pauling to formulate in 1932 a quantitative concept of electronegativity (*X*) based on thermochemistry^4^. Pauling derived values of *X* from bond energies, assuming that extra stabilization of a bond due to its polarization is an additive effect, expressed in electron-volts as

1 $${{D}}_{{\mathrm{AB}}} = {{D}}_{{\mathrm{AB}}}^{{\mathrm{cov}}} + {\Delta}{{X}}_{{\mathrm{AB}}}^2,$$

where *D*_AB_ is the dissociation energy of a single chemical bond between two different atoms A and B, $D_{{\rm{AB}}}^{{\rm{cov}}}$ is the covalent part of that dissociation energy $\left( {{\mathrm{modelled}}\,{\mathrm{as}}\frac{{{{D}}_{{\mathrm{AA}}} + {{D}}_{{\mathrm{BB}}}}}{2}} \right)$, and the amount of stabilization due to the ionic term equals just the electronegativity difference squared. Knowing that fluorine is the most electronegative element and arbitrarily setting its electronegativity to 4, Pauling obtained electronegativities of many elements using formula (1). This thermochemical scale was subsequently refined by Allred^5^, who had more values of dissociation energies at his disposal, and more accurate values than at the time of original Pauling’s paper. The resulting electronegativity scale has become the standard and enjoyed great success, remaining the most popular scale of electronegativity. Traditionally, any new scale, to be taken seriously, had to be consistent with Pauling’s. The additive quadratic form of (1) would allow powerful predictions to be made, for example, for exchange reactions: a reaction AB + CD = AC + BD would be energetically favorable when AC bond is the most polar, and BD bond is the least polar (i.e. electronegativities are *X*_A_ < *X*_B_ < *X*_D_ < *X*_C_). This is related to the hard and soft acids and bases principle^6^. However, it is known that often predictions based on electronegativities fail qualitatively: it is known (and is discussed below) that formula (1) does not work for large electronegativity differences^7,8^, i.e., where its effect is expected to be greatest and most important.

Furthermore, tabulated values of Pauling’s electronegativity for many elements are strange: for example, electronegativities of such metals as Ru, Rh, Pd, Os, Ir, Pt, Au, W, and Mo are higher than the values for B and H, which would imply a positive charge on boron and negative charge on those metal atoms in their borides or hydrides– this is completely counterintuitive. One can also notice a strange dimensionality of Pauling’s electronegativities, eV^1/2^.

Spectroscopic scales of electronegativity are based on data on isolated atoms, among them the Mulliken^9,10^, Allen^11–13^, Martynov and Batsanov^14^, and many other scales. Mulliken electronegativity^9,10^ is defined as the average of the ionization potential and electron affinity. This gives an absolute scale, where electronegativities have a meaningful dimensionality (eV) and have the physical meaning of minus the chemical potential of the electron in an atom, as supported by density functional theory^15–19^, which reinforced the position of Mulliken’s definition. Charge transfer from the less electronegative atom to the more electronegative one can then be viewed as a consequence of equalization of their chemical potentials. The beauty of this scale is counterweighted by difficulties of obtaining electron affinities, which for many elements are still not well known.

Allen^11–13^ proposed another popular spectroscopic scale, where electronegativity is equal to the average energy of valence electrons in a free atom. This approach suffers from an ambiguity as to which electrons should be considered as valence for d- and f-elements. Martynov & Batsanov^14^ used the square root of the average valence ionization energy as a measure of electronegativity, and their electronegativities have the same dimensionality as Pauling’s, i.e., eV^1/2^. Martynov–Batsanov values are very close to Pauling’s, highlighting that completely different definitions converge on the same truth.

Here we reevaluate the concept of electronegativity, which is a key property of the elements expressed many years ago. We identify the drawbacks in the definition of Pauling’s electronegativity scale and reformulate our thermochemical scale on experimental dissociation energies. Our scale displays intuitively correct trends for the 118 elements across the periodic table and reasonably predicts the degrees of ionicity of chemical bonds, improves the separation of elements into metals and non-metals, and greatly improves the description of thermochemistry of molecules and chemical reactions.

## Results and discussion

Let us come back to formula (1) and try to apply it. One can expect the results to be most accurate (greatest signal/noise ratio) at large ${\Delta}{{X}}_{{\mathrm{AB}}}^2$, so we start with alkali and alkali earth fluorides. From experimental bond dissociation energies^20–59^ we find that the ionic stabilization energy is greater in LiF than in CsF or in any alkali fluoride. According to (1) this should indicate that Li is the most electropositive alkali metal and overall electronegativity increases down the group of the periodic table—which is exactly contrary to chemical intuition and to the values one finds in Pauling’s scale. Strangely, Pauling’s electronegativities of alkali metals (and, equally badly, alkali earth metals) cannot be obtained from their highly ionic molecules using Pauling’s formula (1). The values one finds from (1) and experimental bond dissociation energies are *X*(Li) = 1.85, *X*(Na) = 1.96, *X*(K) = 1.99, *X*(Rb) = 2.00, *X*(Cs) = 1.93; these are very different from standard Pauling’s electronegativities of 0.98, 0.93, 0.82., 0.82, and 0.79, respectively (see Supplementary Fig. 1).

The problem is in the form of formula (1): Li–F bond length is much shorter than Cs–F and ionic term should of course be stronger in the shorter bond in Li^+^F^−^ than in Cs^+^F^−^. The same is true for the covalent part of the bond energy, which is also greater in LiF than in CsF. Ionic effects are larger in CsF than in LiF only in relative (relative to covalent effects), but not in absolute sense (see Table 1). This leads to ionic stabilization being not an absolute additive term, but a multiplicative enhancement factor, and the simplest formula is

2 $${{D}}_{{\mathrm{AB}}} = {{D}}_{{\mathrm{AB}}}^{{\mathrm{cov}}} \cdot (1 + {\Delta}{{X}}_{{\mathrm{AB}}}^2)$$

**Table 1:** Bond energetics for alkali fluorides.

Molecule	Dissociation energy ${{D}}_{{\mathrm{AB}}}$, eV	Covalent part ${{D}}_{{\mathrm{AB}}}^{{\mathrm{cov}}}$, eV	Ionic part ${{D}}_{{\mathrm{AB}}}^{{\mathrm{ion}}} = {{D{\!}}_{{\mathrm{AB}}}} - {{D}}_{{\mathrm{AB}}}^{{\mathrm{cov}}}$, eV
Li–F	6.001	1.380	4.621
Na–F	5.379	1.220	4.159
K–F	5.127	1.086	4.041
Rb–F	5.091	1.074	4.017
Cs–F	5.327	1.039	4.288

**Fig. 1:**
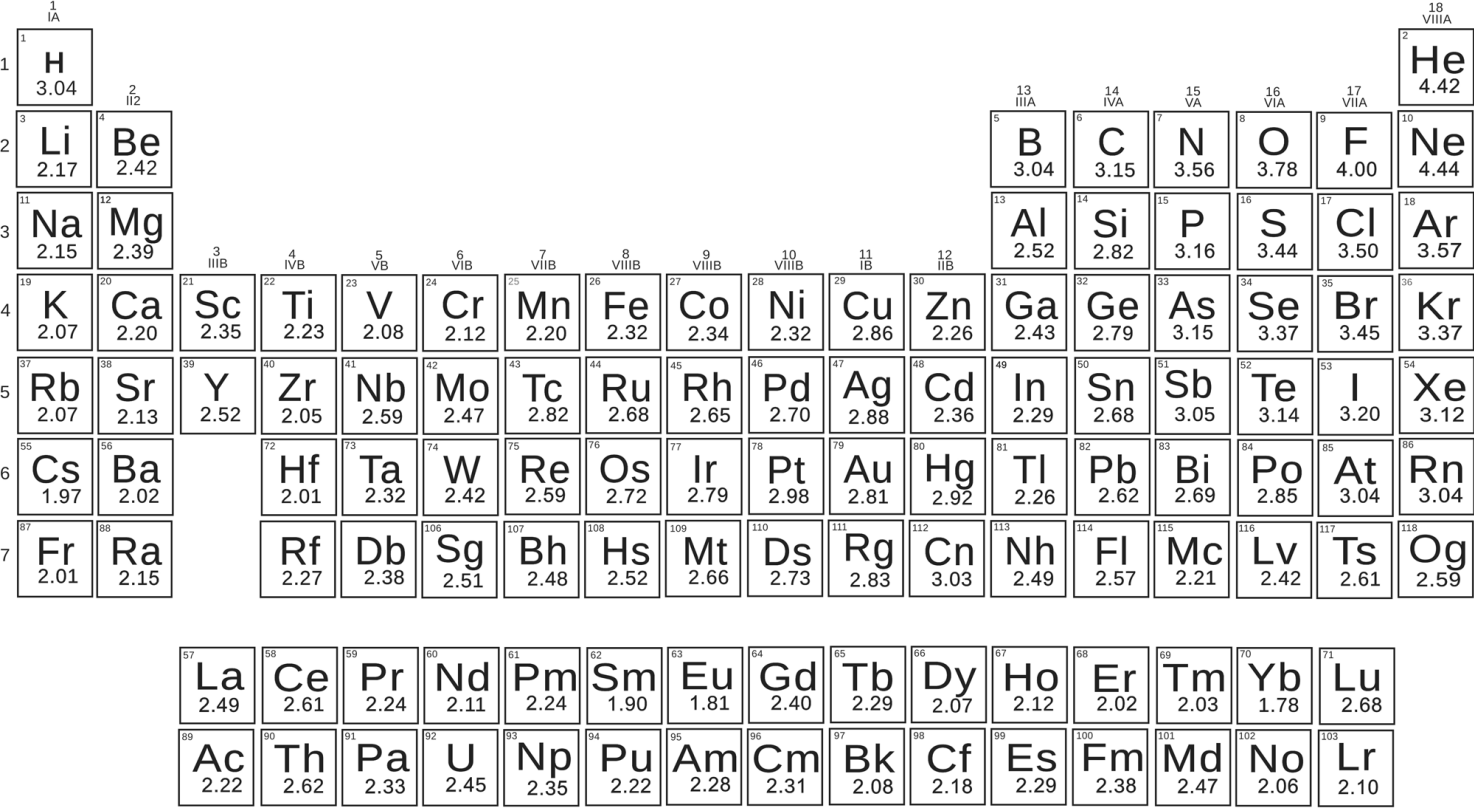
Periodic Table of our thermochemical electronegativities. Electronegativities were obtained using the formula ${{D}}_{{\mathrm{AB}}} = {{D}}_{{\mathrm{AB}}}^{{\mathrm{cov}}} \cdot (1 + {\Delta}{{X}}_{{\mathrm{AB}}}^2)$ (see Eq. 2), where ${{D}}_{{\mathrm{AB}}}$ is the dissociation energy of chemical bond between two different atoms A and B, ${{D}}_{{\mathrm{AB}}}^{{\mathrm{cov}}}$ is the covalent dissociation energy modeled as the arithmetic mean of the homonuclear dissociation energies and ${\Delta}{{X}}_{{\mathrm{AB}}}^2$ is the square of thermochemical electronegative difference between atoms A and B. The electronegativity values of the elements are also provided as Supplementary Data 1.

**Fig. 2:**
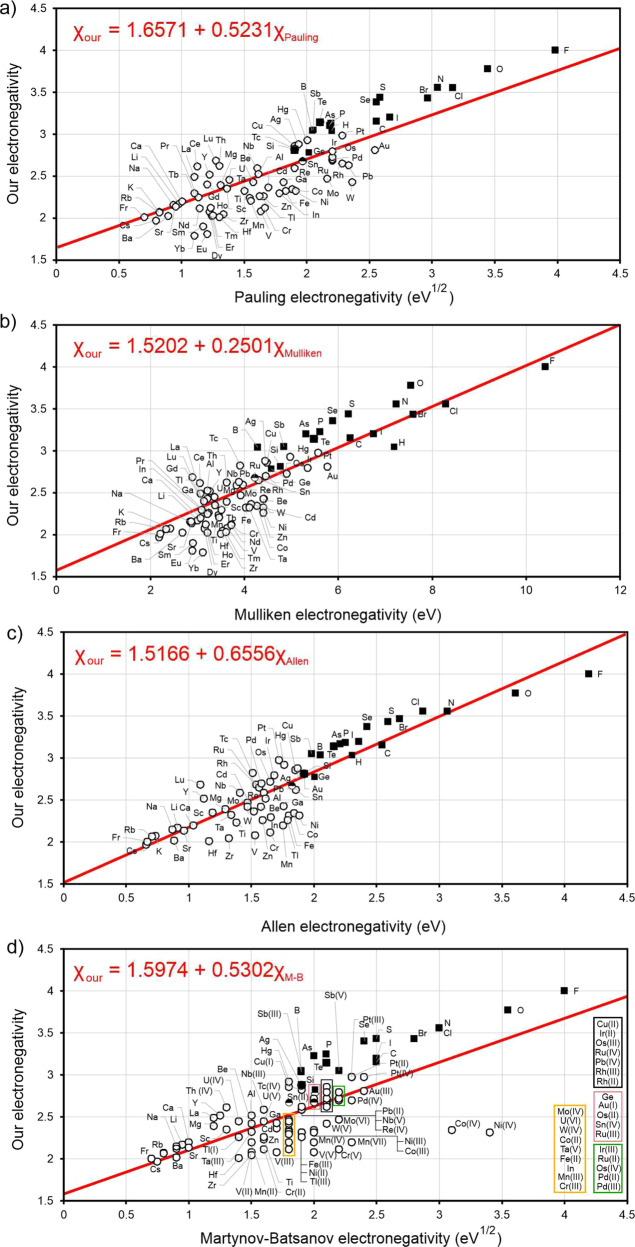
The correlation between our thermochemical electronegativities (*X*_our_) on *y*-axis (dimensionless) with others electronegativities (*x*-axis). **(a)** *X*_our_ vs Pauling (in eV^1/2^), (**b**) *X*_our_ vs Mulliken (in eV), (**c**) *X*_our_ vs Allen (in eV), and (**d**) *X*_our_ vs Martynov-Batsanov (in eV^1/2^). Lines indicate the linear correlation. Legend: metals, empty circles; non-metals, full square.

Now, electronegativities as defined by formula (2) are dimensionless (see Fig. 1). With the help of formula (2) we recover correct trends for the whole periodic table, and do not encounter pathologies such as those mentioned above for alkali and alkali earth metals. All metals have lower electronegativities than boron and hydrogen—in better agreement with chemical intuition than Pauling’s values. Applying formula (2) to molecules ClF, BrF, IF, we obtained electronegativities of Cl (3.56), Br (3.45), I (3.22). Then we recalculated electronegativities of alkali metals using alkali chloride, bromide, and iodide molecules, and found the same values within ~0.2 (which can be considered as uncertainty of our values): for example, the electronegativity of Na extracted from NaF is 2.15, from NaCl 2.28, from NaBr 2.13, from NaI 1.94. Electronegativities of all 118 known elements were calculated using experimental dissociation energies^20–59^ (we took averages of the reported values when two or more measurements were available, see Supplementary Table 2) and are shown in Fig.1. Electronegativities of some elements where there are not enough reliable data on bond energies (noble gases, Pm, Ra, Po, At, and some of the heaviest elements) were obtained indirectly, via a linear correlation with their experimental Mulliken electronegativities, because Mulliken scale shows the best correlation with our scale. In fact, our thermochemical scale has reasonable linear correlation with all other scales (Fig. 2 and Supplementary Table 3), Pearson correlation coefficient R being 98% for Mulliken and Allen scales, 87% for Pauling, and 85% for Martynov–Batsanov scales). For short-lived 6d- and 7p-elements Rf, Db, Sg, Bh, Hs, Mt, Ds, Rg, Cn, Nh, Fl, Mc, Lv, Ts, Og we obtained thermochemical electronegativities from theoretical Mulliken electronegativities^60,61^. When more data become available for these elements, then thermochemical electronegativities will be determined directly. Interestingly, the slope of the correlation line is 1/2 for Pauling’s and Martynov–Batsanov scales, 2/3 for Allen’s and 1/4 for Mulliken’s scale. One can see the expected trends in the periodic table: periodicity of electronegativities, and their overall decrease from top to bottom of the table. The highest electronegativities are those of halogens and noble gases; the lowest—of alkali metals (Li: 2.17, Cs: 1.97) and, surprisingly, some other metals (Zr: 2.05, Hf: 2.01, and two anomalous lanthanoids are even slightly lower than Cs–Eu: 1.81, Yb: 1.78). For the non-alkali anomalous metals, low values are consistent with their tendency to react highly exothermically with oxygen and fluorine; Yb even vigorously reacts with water. It is also known that Eu and Yb (and to a lesser extent Sm) display chemical behavior which is different from the other lanthanoids, preferring divalent state. From the viewpoint of physical properties, Yb has more than two times higher electrical conductivity than the other lanthanoids, and both Eu and Yb have work functions which are lower than those of the other lanthanoids and among the lowest in the periodic table (2.5 and 2.6 eV, respectively—compared with 2.9 eV for Li, 2.36 eV for Na, and 3.5 eV for La^62^).

Electronegativity can be used as a criterion to discriminate between metals and non-metals, but different scales do so with different degrees of success. The best separation into metals and non-metals is achieved with our and Allen’s scales. For example, all elements with electronegativity above 3 (in our scale) are non-metals. Almost all elements with electronegativity below 3 are metals; the few exceptions are Si (2.82), Ge (2.79), Sn (2.68, but Sn is known at normal conditions in the metallic white tin and semiconducting gray tin allotropes). The scales of Pauling, Mulliken and Martynov–Batsanov work well too, but have difficulties assigning noble metals and a few other elements. From our table of electronegativities (Fig. 1), one can expect oganesson (Og, element #118, belonging to the group of noble gases) to be much more reactive than noble gases: with electronegativity as low as 2.59, it is expected to be a metal similar to Pb (electronegativity 2.62). Such low electronegativity comes from relativistic effects, which are particularly strong in superheavy elements, and non-inertness of Og is consistent with suggestions from literature^63–65^. By contrast, due to relativistic stabilization of its valence 7s^2^ shell, copernicium (Cn, element #112), belonging to the same group as mercury, has an anomalously high electronegativity of 3.03, similar to radon (electronegativity 3.04), non-metallic and rather inert element. Due to relativistic effects, some superheavy elements display unexpected similarity to other groups of the periodic table^66,67^.

**Fig. 3:**
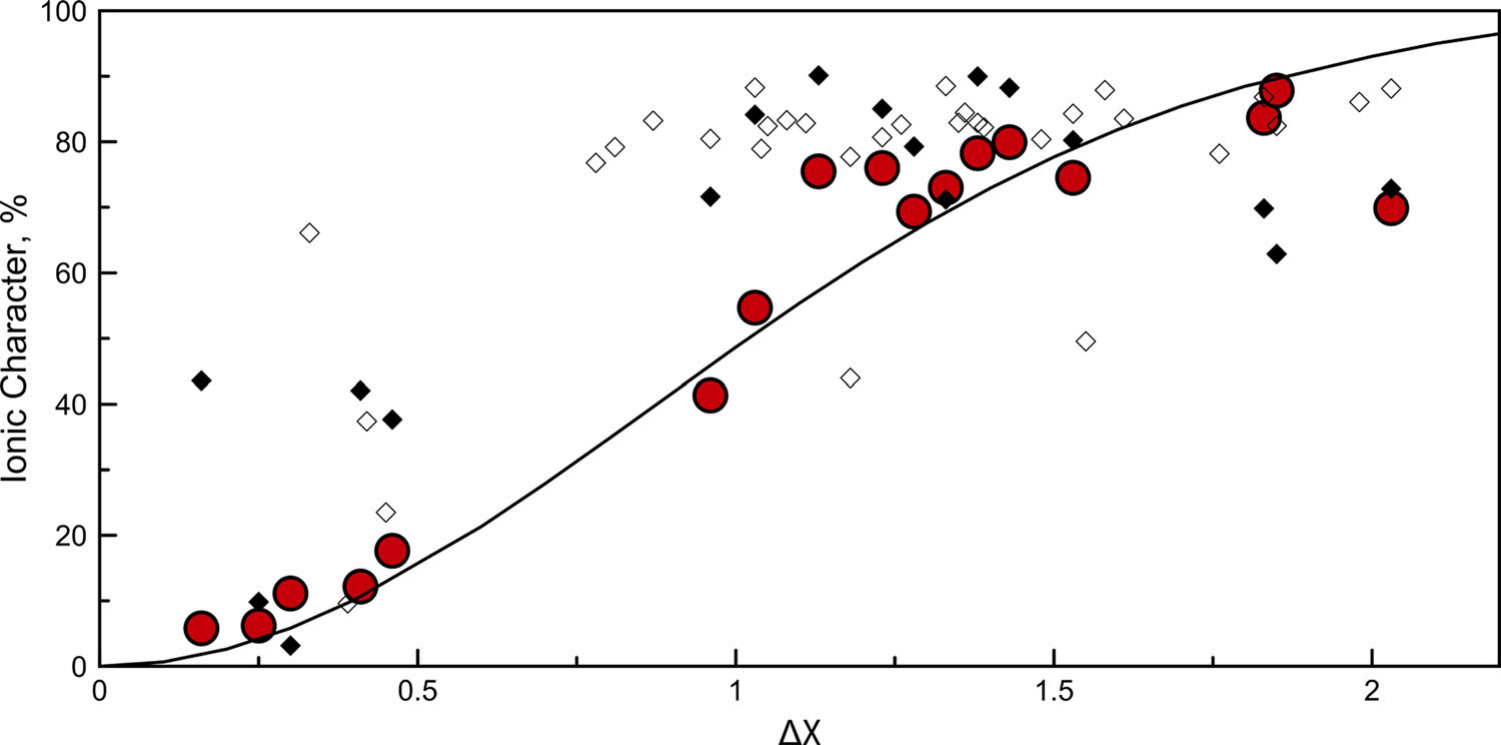
Ionic character in diatomic molecules. Bond ionic character (IC) for different compounds calculated from dipole moment (i.e., red circles) and Bader charges for molecules (i.e., filled diamonds) and crystals (i.e., open diamonds) vs electronegativity difference (our scale).

Pauling^4^ argued that extra stabilization of a bond (see formulae (1) and (2)) is due to a resonance mixing of covalent and ionic wavefunctions, the resulting charge asymmetry being determined by electronegativity difference. Pauling proposed to estimate the degree of ionicity by the heuristic formula:

3 $${{f}}\left( {{\Delta}{{X}}} \right) = 1 - {\mathrm{e}}^{ - {{k}} \cdot {\Delta}{{X}}^2}$$

where *k* = 0.25. This function was calibrated to describe experimental dipole moments of a number of diatomic molecules. Using our electronegativities, we can describe the same data with *k* = 0.67 (see Fig. 3) and achieving at least the same accuracy (RMSD is 9.5% vs 9.8%). The same is true for ionicity degrees obtained from Bader charges^68^ (see Supplementary Table 4–6), although with these charges the scatter is much greater (RMSD is 25.0% for our predictions vs 30.1 % for predictions based on Pauling’s formula).

We see that our electronegativity scale leads to correct bond polarity even where Pauling’s scale fails. For example, Pauling electronegativities of Ru, Rh, Pd, Os, Ir, Pt, Au, W, and Mo are higher than those of H and B, and one would obtain negative charges on metal atoms in their borides and hydrides. In our scale the opposite is the case, which agrees with the calculated Bader charges: e.g., W^+0.69 ^B^−0.69^, Mo^+0.61 ^B^−0.61^, Au^+0.14 ^B_2_^−0.07^, Pt^+0.24 ^H_4_^−0.06^.

There is some physical difference between our use of function (4) and Pauling’s. In our case, the squared electronegativity difference in the exponent in (4) is the ratio of the ionic and covalent contributions to bond energy. This should be more directly related to the degree of ionicity than just the ionic contribution taken without regard for the covalent energy (as in Pauling’s version).

**Fig. 4:**
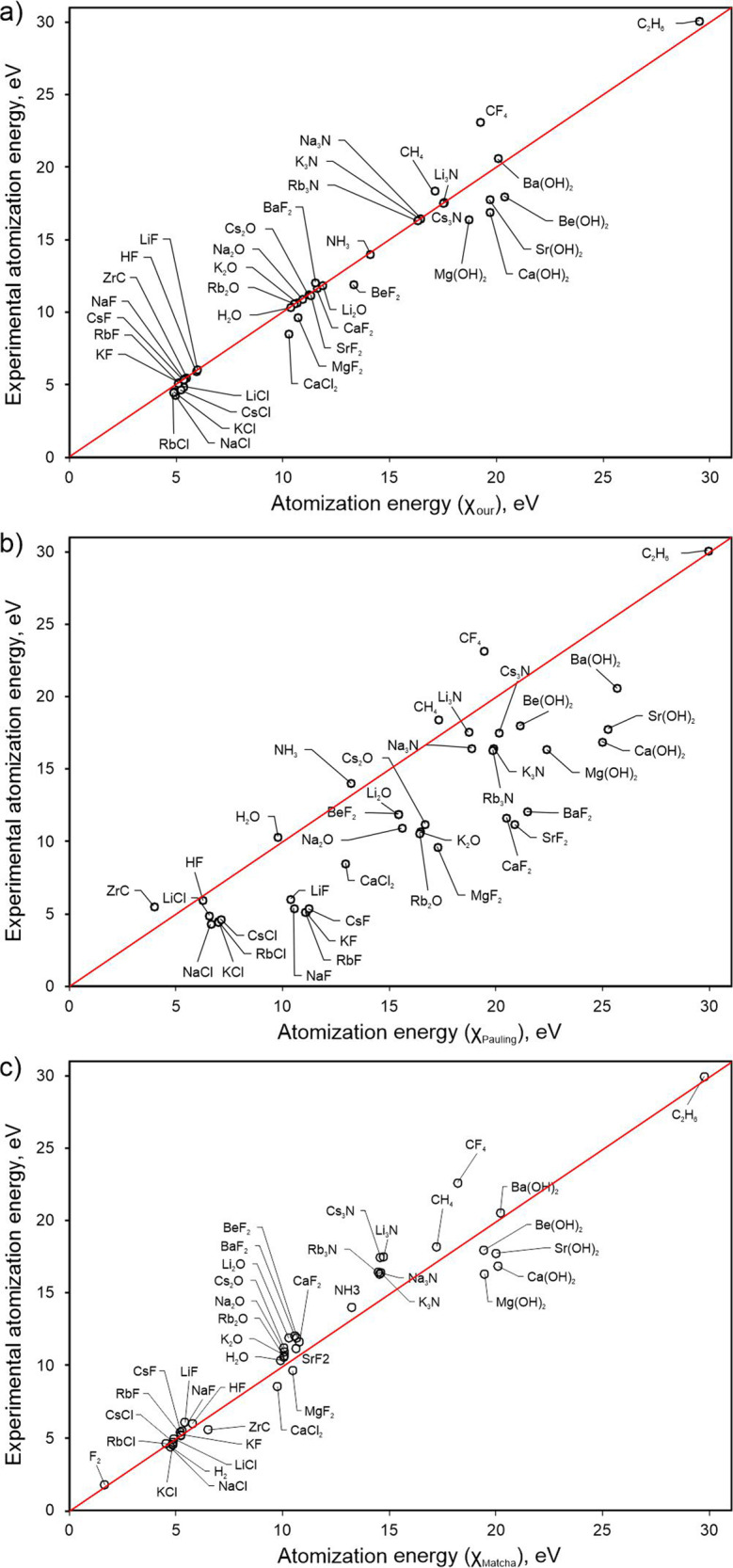
Atomization energies predicted from thermochemical electronegativities (*x*-axis) for a number of simple molecules in comparison with experimental atomization energies on *y*-axis. (**a**) Experimental vs our, (**b**) experimental vs Pauling’s (**c**) and experimental vs Pauling’s corrected by Matcha ^8^. Lines indicate the ideal results, the root-mean-square deviations from which are 0.17 eV/atom for our approach, 1.21 eV/atom for Pauling’s, and 0.25 eV/atom for Matcha’s (the relative errors on atomization energies are 5%, 40, and 7%, respectively).

Derived from thermochemistry, our electronegativity scale should be capable of at least qualitatively correctly predicting the outcome of chemical reactions, heats of formation and atomization energies of molecules and solids. To correct deficiencies of Pauling’s formula (1), Matcha^8^ introduced another formula (with energies in eV):

4 $${{D}}_{{\mathrm{AB}}} = {{D}}_{{\mathrm{AB}}}^{{\mathrm{cov}}} + {{K}}\left[ {1 - {\mathrm{exp}}\left( { - {\Delta}{{X}}^2/{{K}}} \right)} \right]$$

with the adjustable parameter *K* = 4.56 eV. Let us illustrate how our results compare with Pauling’s and Matcha’s results. Using our electronegativities from Fig. 1 and energies of single homonuclear bonds X–X from Supplementary Table 2, we estimated the atomization energy of ethanol, C_2_H_5_OH, as the sum of 5 C–H bonds, 1 C–C bond, 1 C–O bond, and 1 O–H bond. Neglecting the ionic term in (2), we obtain 31.37 eV—while including it we get 34.67 eV, just 2% off the experimental result (33.94 eV^59^). The results from Pauling’s formula (1) and from Matcha’s formula (4) are 34.32 and 33.64 eV, respectively. For a more ionic molecule as NaCl our estimation of the atomization energy is 4.81 eV and predictions based on Pauling’s and Matcha’s approaches are 6.58 eV and 4.71 eV, respectively, to compare with experiment (4.29 eV^59^). Clearly, both Matcha’s and our approaches greatly improve upon Pauling’s approach. We have extended such comparison to a set of molecules (fluorides, oxides, hydroxides, chlorides, nitrides, hydrides, carbides) with various degrees of ionicity, and compared their predicted atomization energies with experiment (see Fig. 4). Overall, our scale achieves much more accurate predictions than Pauling’s scale and is also more accurate than Matcha’s approach. These advantages become greater when one looks at energies of reactions, i.e., energy differences.

The same approach can be used for estimating the enthalpies of the formation of compounds. Taking the NaCl molecule again as example, we predict the energy of the reaction of its formation in the gas phase (Na_2_ + Cl_2_ = 2NaCl) as −6.31 eV using our electronegativity scale, and as −9.85 eV from Pauling’s approach and −9.42 eV from Matcha’s approach; clearly, the estimation based on our electronegativities is much closer to experiment (−5.27 eV from experimental energies of molecules^59^). Large negative value indicates that the formation of NaCl from the elements is highly favorable.

Thermochemical electronegativities should be capable of predicting the direction of at least simple chemical reactions. It is known that Pauling’s electronegativities often lead to incorrect predictions^12^. Let us take the reaction:

5 $$2{\mathrm{NaF}} + {\mathrm{CaCl}}_2 = 2{\mathrm{NaCl}} + {\mathrm{CaF}}_2$$

**Fig. 5:**
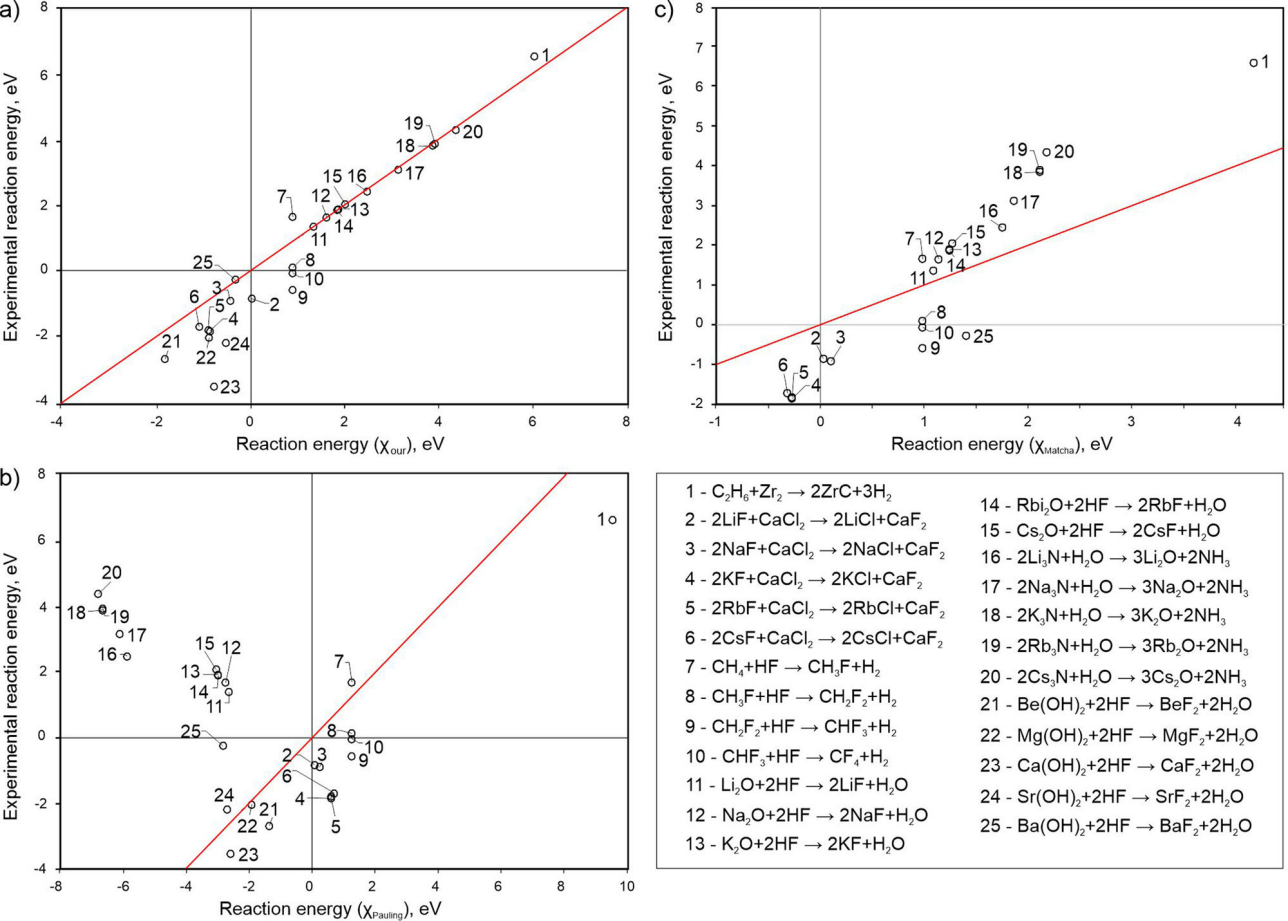
Comparison between predicted energies of 25 exchange reactions from thermochemical electronegativities (*x*-axis) and and experimental energies on *y*-axis. (**a**) Experimental vs our, (**b**) experimental vs Pauling’s (**c**) and experimental vs Pauling’s corrected by Matcha ^8^. Lines indicate the ideal result (the root-mean-square deviations are equal to 0.9 eV for our approach, 5.1 eV for Pauling’s and 1.9 eV for Matcha’s).

Ignoring the ionic term, one would find that the enthalpy of this reaction is zero. Pauling’s electronegativities and formula (1) give a positive value, +0.23 eV, incorrectly predicting that this reaction is unfavorable. Matcha’s approach gives +0.11 eV. Our electronegativity scale and formula (2) show that this molecular reaction is favorable, with enthalpy −0.45 eV. The experimental value is −0.92 eV^59^. Figure 5 shows energies of very different exchange reactions (from reaction (3) to hydrolysis of Li_3_N and fluorination of methane) calculated using electronegativities and using experimental molecular energies. One can see that in virtually all cases our model predicts the correct sign and reasonable magnitude of the reaction energy, in contrast to predictions based on Pauling’s approach. Our approach is also clearly much more accurate than Matcha’s.

Electronegativity is expected to correlate with many physical properties of materials—from mechanical (such as hardness, see—Ref. ^69,70^) to electronic, optical etc. We showed above how well it discriminates between metals and non-metals. It can be expected to correlate with the work function, which (just like Mulliken’s electronegativity for an isolated atom) is equal to the chemical potential of the electron on the surface. This link has been known before^71,72^, although the correlation is not perfect (see Supplementary Fig. 7): the best correlation coefficient (91%) is for Pauling’s scale, followed by Mulliken’s (83%), Martynov-Batsanov (79%), our (65%) and Allen’s (63%) scales, because o effects of the crystal structure (which lead to broadening of valence electron energy levels) and of the surface (the work function varies significantly between different surfaces of the same material).

To sum up, we have shown how a simple modification of the definition of thermochemical electronegativity leads to a greatly improved electronegativity scale. Our electronegativities are dimensionless (instead of unusual units eV^1/2^ of Pauling’s electronegativities), display intuitively correct trends across the periodic table, allow for reasonable prediction of bond polarity and degree of ionicity, improve the separation of elements into metals and non-metals, and, most importantly, greatly improve the description of thermochemistry of molecules and chemical reactions. We expect our scale of electronegativity to find widespread use in chemistry and physics.

## Methods

### Computational details

Bader charges were calculated for crystal structures (see Supplementary Table 4) taken from Materials Project^73^ and fully reoptimized using first-principle calculations performed with ab-initio total-energy and molecular-dynamics program VASP (Vienna ab-initio simulation program).^74^ For such calculations PBE^75,76^ exchange-correlation functional and PAW^77^ method. The kinetic energy cut-off was set to 1000 eV and the threshold of electron energy and forces were both set in the order of 1e-8 (eV/cell for the energy and eV/atom for the forces). Bader analysis was performed using the Yu–Trinkle algorithm^78^ on total electron densities obtained on fully relaxed structures.

## Supplementary information

Supplementary Information

Description of Additional Supplementary Files

Supplementary Data 1

## Data Availability

All relevant data are included in the paper and its supplementary information files.
