# Author Correction: Thermochemical electronegativities of the elements

**DOI:** 10.1038/s41467-021-23670-3

**Published:** 2021-05-28

**Authors:** Christian Tantardini, Artem R. Oganov

**Affiliations:** 1 Skolkovo Institute of Science and Technology, Bolshoi Boulevard 30, Moscow, 121025 Russian Federation; 2 Institute of Solid State Chemistry and Mechanochemistry SB RAS, 630128 Kutateladze 18, Novosibirsk, Russian Federation

**Keywords:** History of chemistry, Chemical bonding, Method development

Correction to: *Nature Communications* 10.1038/s41467-021-22429-0, published online 7 April 2021.

The original version of this Article contained several errors.

The fourth sentence of the Abstract incorrectly read ‘electronegativities have units of eV^−1/2^’; the second sentence of the second paragraph of the Introduction incorrectly read ‘Pauling’s electronegativities, eV^−1/2^’; the third sentence of the fourth paragraph of the Introduction incorrectly read ‘the same dimensionality as Pauling’s, i.e., eV^−1/2^’; the second sentence of the thirteenth paragraph of the ‘Results and discussion’ section incorrectly read ‘instead of unusual units eV^−1/2^ of Pauling’s electronegativities’; the first sentence in the legend of Fig. 2 incorrectly read ‘X_our_ vs Pauling (in eV^−1/2^)’ and ‘*X*_our_ vs Martynov-Batsanov (in eV^−1/2^)’; the X axis label of Figure 2 incorrectly read ‘Martynov-Batsanov electronegativity (eV^−1/2^)’. The correct version states ‘eV^1/2^’ in place of ‘eV^−1/2^’ at all of these locations.

The first sentence in the legend of Fig. 1 incorrectly read ‘modeled as the arithmetic difference between the homonuclear dissociation energy’. The correct version states ‘mean of the homonuclear dissociation energies’ in place of ‘difference between the homonuclear dissociation energy’.

The second sentence in the legend of Fig. 2 incorrectly read ‘Lines indicate the linear interpolation function’. The correct version states ‘correlation’ in place of ‘interpolation function’.

The first sentence in the legend of Fig. 3 incorrectly read ‘Δ*X* of our electronegativity’. The correct version states ‘electronegativity difference (our scale)’ in place of ‘Δ*X* of our electronegativity’.

The third and fourth sentences of the third paragraph in the Introduction incorrectly read ‘This gives an absolute scale, where electronegativities have a meaningful dimensionality (eV) and have the physical meaning of minus the chemical potential of the electron in an atom^15–19^. The position of Mulliken’s electronegativity was reinforced in the last years by the cornerstones of density functional theory being correlated to the chemical potential by the second Hohenberg-Kohn theorem^15–19’^. The correct version states ‘This gives an absolute scale, where electronegativities have a meaningful dimensionality (eV) and have the physical meaning of minus the chemical potential of the electron in an atom, as supported by density functional theory^15–19^, which reinforced the position of Mulliken’s definition’.

The fifth sentence of the third paragraph of the ‘Results and discussion’ section incorrectly read ‘we recalculated electronegativities of alkali metals using alkali chlorides, bromides and iodides’. The correct version states ‘chloride, bromide, and iodide molecules’ instead of ‘chlorides, bromides and iodides’.

The seventh sentence of the eight paragraph of the ‘Results and discussion’ section incorrectly read ‘much further from experiment’. The correct version states ‘to compare with’ instead of ‘much further from’.

The first sentence of the ‘Computational details’ section of the Methods incorrectly read ‘The crystal structures of taken into account systems (see Supplementary Table 4) were found in Materials Project’. The correct version replaces this sentence with ‘Bader charges were calculated for crystal structures (see Supplementary Table 4) taken from Materials Project’.

This has been corrected in both the PDF and HTML versions of the Article.

