# Author Correction: Thermochemical electronegativities of the elements

**DOI:** 10.1038/s41467-021-24655-y

**Published:** 2021-07-14

**Authors:** Christian Tantardini, Artem R. Oganov

**Affiliations:** 1 Skolkovo Institute of Science and Technology, Bolshoi Boulevard 30, Moscow, 121025 Russian Federation; 2 Institute of Solid State Chemistry and Mechanochemistry SB RAS, 630128, Kutateladze 18, Novosibirsk, Russian Federation

**Keywords:** History of chemistry, Chemical bonding, Method development

Correction to: *Nature Communications* 10.1038/s41467-021-22429-0, published online 7 April 2021.

The original version of this Article contained two errors in Figure 2, in which the elements silicon and tellurium were represented as metals (empty circles) instead of non-metals (full squares). This has been corrected both in the PDF and in the HTML versions of the Article.

