# Supplementary material for: Thermochemical electronegativities of the elements
Source: Nat Commun. 2021 Apr 7;12:2087. doi: 10.1038/s41467-021-22429-0 (PMC8027013; doi:10.1038/s41467-021-22429-0)
Supplement: Supplementary file 1 — Supplementary Information [file 41467_2021_22429_MOESM1_ESM.pdf]

Supplementary Information

Thermochemical Electronegativities of the Elements

Christian Tantardini*[1,2], Artem R. Oganov*[1]

[1]*Skolkovo Institute of Science and Technology, Skolkovo Innovation Center 121025, 3 Nobel Street, Moscow, Russia*

[2]*Institute of Solid State Chemistry and Mechanochemistry SB RAS, 630128 Novosibirsk, Russian Federation.*

**corresponding authors*: christiantantardini@ymail.com, A.Oganov@skoltech.ru

Supplementary Figure 1. Standard Pauling electronegativities (x-axis, in $eV^{-1/2}$) compared with Pauling electronegativities (y-axis, in $eV^{-1/2}$) calculated using experimental bond energies for alkali and alkali earth metals. One can see very large difference.

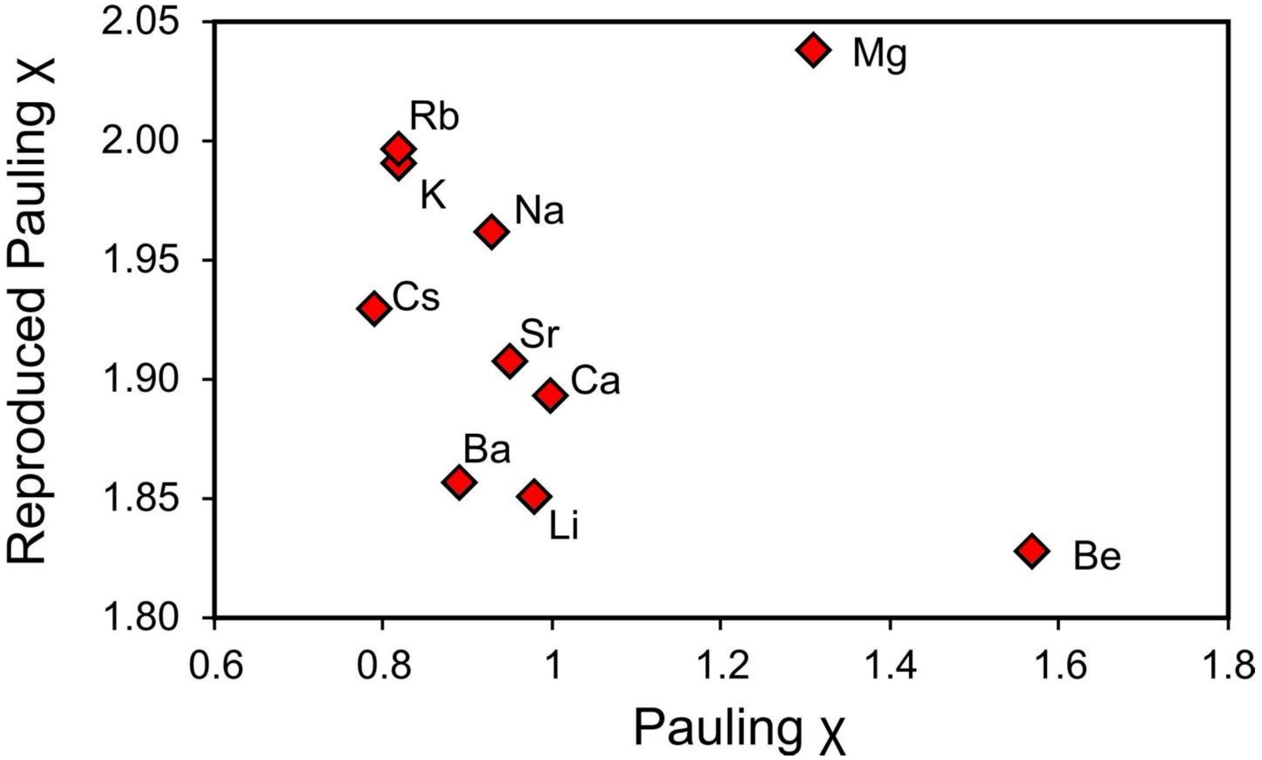

Supplementary Table 2. Experimental bond dissociation energies used for calculating electronegativities.

Homonuclear bonds	Dissociation Enthalpy / (eV)	Heteronuclear bonds	Dissociation Enthalpy / (eV)
H2	4.517	HF	5.904
Li2	1.116	LiF	6.001
Be2	2.156	BeF	6.622
B2	4.353	F2B-F	5.773
C2H6 (C-C)	3.911	H3C-F	4.770
N2H4 (N-N)	2.871	H2N-H	4.665
H2O2 (O-O)	2.183	H-OH	5.152
F2	1.644		
Na2	0.796	NaF	5.379
Mg2	1.337	MgF	5.343
Al2	2.739	AlF	6.996
H3Si-SiH3 (Si-Si)	3.327	SiF	5.974
H2P-PH2 (P-P)	2.654	H2P-H	3.638
FS-SF (S-S)	3.755	SF	3.560
Cl2	2.514	HCl	4.471
K2	0.528	KF	5.127
Ca2	1.088	CaF	5.807
Sc2	1.689	ScF	6.209
Ti2	1.219	TiF	5.897
V2	0.961	VF	6.115
Cr2	0.742	CrF	5.421
Mn2	0.532	MnF	4.614
Fe2	0.778	FeF	4.633
Co2	0.740	CoF	4.467

Ni2	0.741	NiF	4.557
Cu2	2.083	CuF	4.291
Zn2	0.230	ZnF	3.773
Ga2	1.197	GaF	4.941
Ge2	2.740	GeF	5.421
As2	3.999	AsF	4.249
Se2	1.769	SeF	3.513
Br2	2.056	FBr	2.637
Rb2	0.503	RbF	5.091
Sr2	0.871	SrF	5.638
Y2	2.798	YF	7.103
Zr2	1.052	ZrF	6.500
Nb2	1.349	NbF	4.480
Mo2	1.227	MoF	4.809
Tc2	0.050	TcF	2.020
Ru2	1.125	RuF	3.781
Rh2	0.963	RhF	3.686
Pd2	0.644	PdF	3.088
Ag2	1.631	AgF	3.699
Cd2	0.076	CdF	3.161
In2	1.036	InF	5.244
Sn2	1.939	SnF	4.933
Sb2	3.127	SbF	4.550
Te2	2.860		
I2	1.681	FI	2.730
Cs2	0.433	CsF	5.327
Ba2	0.698	BaF	5.767

Hf2	1.070	HfF	6.737
Ta2	1.469	TaF	5.939
W2	1.594	WF	5.638
Re2	1.340	ReF	4.458
Os2	1.364	OsF	3.955
Ir2	1.149	IrF	3.430
Pt2	0.980	PtF	2.684
Au2	2.294	AuCl	3.555
Hg2	0.084	HgF	1.866
Tl2	0.616	TlF	4.550
Pb2	0.898	PbF	3.679
Fr2	0.430	FrF	5.160
La2	2.520	LaF	6.830
Ce2	2.470	CeF	6.032
Pr2	1.310	PrF	6.032
Nd2	0.830	NdF	5.651
Sm2	0.520	SmF	5.856
Eu2	0.300	EuF	5.638
Gd2	1.780	GdF	6.115
Tb2	1.320	TbF	5.814
Dy2	0.690	DyF	5.503
Ho2	0.820	HoF	5.597
Er2	0.740	ErF	5.856
Tm2	0.520	TmF	5.286
Yb2	0.170	YbF	5.365
Lu2	1.430	LuF	4.198
Th2	2.995	ThF	6.758

U2	2.301	UF	6.716
-	-	LiCl	4.710
-	-	NaCl	4.384
-	-	KCl	4.405
-	-	RbCl	4.474
-	-	CsCl	4.619
-	-	LiBr	4.195
-	-	NaBr	3.900
-	-	KBr	3.929
-	-	RbBr	3.946
-	-	CsBr	4.033
-	-	LiI	3.534
-	-	NaI	3.196
-	-	KI	3.343
-	-	RbI	3.304
-	-	CsI	3.508

Supplementary Table 3. Electronegativities of the elements from different scales. For Martinov-Batsanov scale there are different electronegativity values for the different oxidation states (reported in brackets) of each element.

Element	Pauling / ($eV^{-1/2}$)	Mulliken / (*eV*)	Allen / (*eV*)	Martynov & Batsanov / ($eV^{-1/2}$)	Our scale
H	2.20	7.18	2.3	-	3.04
Li	0.98	3.00	0.912	0.95	2.17
Na	0.93	2.84	0.869	0.9	2.15
K	0.82	2.42	0.734	0.8	2.07
Rb	0.82	2.33	0.706	0.8	2.07
Cs	0.79	2.18	0.659	0.75	1.97
Fr	0.70	2.21	0.67	0.7	2.01
Be	1.57	4.41	1.576	1.5	2.42
Mg	1.31	3.62	1.293	1.2	2.39
Ca	1.00	3.07	1.034	1	2.20
Sr	0.95	2.87	0.963	1	2.13
Ba	0.89	2.68	0.881	0.9	2.02
Ra	0.90	2.69	0.89	0.9	-
Sc	1.36	3.37	1.19	1.3	2.35
Ti	1.54	3.45	1.38	1.6	2.23
V	1.63	3.64	1.53	(II) 1.5 (III) 1.7 (V) 2.00	2.08
Cr	1.66	3.72	1.65	(II) 1.6 (III) 1.8 (V) 2.2	2.12
Mn	1.55	3.46	1.75	(II) 1.5 (III) 1.8 (IV) 2.0 (VII) 2.3	2.20
Fe	1.83	4.03	1.8	(II) 1.8 (III) 1.9	2.32
Co	1.88	4.27	1.84	(II) 1.8 (III) 2.0 (IV) 3.1	2.34
Ni	1.91	4.40	1.88	(II) 1.9 (III) 2.0 (IV) 3.4	2.32
Cu	1.90	4.48	1.85	(I) 1.8) (II) 2.1	2.86
Zn	1.65	4.40	1.59	1.6	2.26
Y	1.22	3.26	1.12	1.25	2.52
Zr	1.33	3.53	1.32	1.5	2.05
Nb	1.60	3.84	1.41	(III) 1.6 (V) 1.9	2.59
Mo	2.16	3.92	1.47	(IV) 1.8 (VI) 2.2	2.47
Tc	1.90	3.91	1.51	(IV) 1.9	2.82

Ru	2.20	4.20	1.54	(II) 2.0 (III) 2.0 (IV) 2.1	2.68
Rh	2.28	4.30	1.56	(II) 2.1 (III) 2.1	2.65
Pd	2.20	4.45	1.58	(II) 2.2 (III) 2.2 (IV) 2.3	2.70
Ag	1.93	4.44	1.87	1.9	2.88
Cd	1.69	4.14	1.52	1.7	2.36
Hf	1.30	3.50	1.16	1.4	2.01
Ta	1.50	4.10	1.34	(III) 1.5 (V) 1.8	2.32
W	2.36	4.40	1.47	(IV) 1.8 (V) 2.1	2.42
Re	1.90	3.97	1.6	(IV) 1.9	2.59
Os	2.20	4.89	1.65	(II) 2.0 (III) 2.1 (IV) 2.2	2.72
Ir	2.20	5.34	1.68	(II) 2.1 (III) 2.2	2.79
Pt	2.28	5.57	1.72	(II) 2.3 (III) 2.3 (IV) 2.4	2.98
Au	2.54	5.77	1.92	(I) 2.0 (III) 2.4	2.81
Hg	2.00	4.97	1.76	1.8	2.92
B	2.04	4.29	2.05	1.9	3.04
Al	1.61	3.21	1.613	1.5	2.52
Ga	1.81	3.21	1.756	1.7	2.43
In	1.78	3.09	1.656	1.8	2.29
Tl	1.62	3.24	1.789	(I) 1.4 (III) 1.9	2.26
C	2.55	6.26	2.544	2.5	3.15
Si	1.90	4.77	1.916	1.9	2.82
Ge	2.01	4.57	1.994	2.0	2.79
Sn	1.96	4.23	1.824	(II) 1.8 (IV) 2.0	2.68
Pb	2.33	3.89	1.854	(II) 1.9 (IV) 2.1	2.62
N	3.04	7.23	3.066	3.0	3.56
P	2.19	5.62	2.253	2.1	3.16
As	2.18	5.31	2.211	2.0	3.15
Sb	2.05	4.85	1.984	(III) 1.9 (V) 2.2	3.05
Bi	2.02	4.11	2.01	(III) 1.9 (V) 2.2	-
O	3.44	7.54	3.61	3.55	3.78
S	2.58	6.22	2.589	2.5	3.44
Se	2.55	5.89	2.424	2.4	3.37

Te	2.10	5.49	2.158	2.1	3.14
Po	2.00	4.91	2.19	2.0	-
F	3.98	10.41	4.193	4.0	4.00
Cl	3.16	8.29	2.869	3.0	3.56
Br	2.96	7.59	2.685	2.8	3.45
I	2.66	6.76	2.359	2.5	3.20
At	2.20	5.87	2.39	2.2	-
La	1.10	3.06	-	1.2	2.49
Ce	1.12	3.05	-	-	2.61
Pr	1.13	3.21	-	-	2.24
Nd	1.14	3.72	-	-	2.11
Pm	1.13	2.86	-	-	-
Sm	1.17	2.90	-	-	1.90
Eu	1.20	2.89	-	-	1.81
Gd	1.20	3.14	-	-	2.40
Tb	1.10	3.51	-	-	2.29
Dy	1.22	3.15	-	-	2.07
Ho	1.23	3.18	-	-	2.12
Er	1.24	3.21	-	-	2.02
Tm	1.25	3.61	-	-	2.03
Yb	1.10	3.12	-	-	1.78
Lu	1.27	2.89	1.09	-	2.68
Th	1.30	3.63	-	(IV) 1.3	2.62
U	1.38	3.36	-	(IV) 1.4 (V) 1.6 (VI) 1.8	2.45
He	-	12.29	4.16	-	-
Ne	-	10.78	4.787	-	-
Ar	-	7.88	3.242	-	-
Kr	3.23	7.00	2.966	-	-
Xe	3.02	6.07	2.582	-	-
Rn	2.81	5.37	2.6	-	-

Supplementary Table 4. Lattice parameters of the crystal structures used for Bader charge calculations.

System	Space Group	a / (Å)	b / (Å)	c / (Å)	α	β	γ
Al_2O_3	$R\underline{3}c$	4.803	4.803	13.110	90.00	90.00	120.00
AlN	$P6_3mc$	3.127	3.127	5.013	90.00	90.00	120.00
AuB_2	$P6/mmm$	3.005	3.005	4.281	90.00	90.00	120.00
MoB	$I4_1/amd$	3.127	3.127	17.007	90.00	90.00	90.00
WB	$I4_1/amd$	3.139	3.139	16.947	90.00	90.00	90.00
Bi_2Te_3	$R\underline{3}m$	4.444	4.444	31.484	90.00	90.00	120.00
LiH	$Fm\underline{3}m$	2.841	2.841	2.841	60.00	60.00	60.00
PtH_4	$Im\underline{3}m$	6.711	6.711	6.711	90.00	90.00	90.00
PbH	$P6_3mc$	3.514	3.514	7.369	90.00	90.00	120.00
RhH	$Fm\underline{3}m$	4.043	4.043	4.043	90.00	90.00	90.00
BaO	$Fm\underline{3}m$	5.601	5.601	5.601	90.00	90.00	90.00
BeO	$P6_3mc$	2.702	2.702	4.389	90.00	90.00	120.00
MgO	$Fm\underline{3}m$	4.248	4.248	4.248	90.00	90.00	90.00
LiF	$Fm\underline{3}m$	4.072	4.072	4.072	90.00	90.00	90.00
LiCl	$Fm\underline{3}m$	3.643	3.643	3.643	60.00	60.00	60.00
LiI	$P6_3mc$	4.261	4.261	4.261	60.00	60.00	60.00
NaF	$Fm\underline{3}m$	3.321	3.321	3.321	90.00	90.00	90.00
NaCl	$Fm\underline{3}m$	5.696	5.696	5.696	90.00	90.00	90.00
NaI	$Fm\underline{3}m$	6.532	6.532	6.532	90.00	90.00	90.00
CsF	$Fm\underline{3}m$	4.326	4.326	4.326	60.00	60.00	60.00
CsCl	$Fm\underline{3}m$	7.087	7.087	7.087	90.00	90.00	90.00
CsI	$Fm\underline{3}m$	5.557	5.557	5.557	60.00	60.00	60.00
BeF_2	$I\underline{4}3m$	8.561	8.561	8.561	90.00	90.00	90.00
$BeCl_2$	$Ibam$	5.247	5.738	6.586	115.82	113.47	90.00
BeI_2	$Ibam$	6.505	6.143	7.554	113.99	115.50	90.00
MgF_2	$Ibam$	3.094	4.686	4.703	90.00	90.00	90.00
$MgCl_2$	$R\underline{3}m$	6.908	6.908	6.908	30.86	30.86	30.86
MgI2	$P\underline{3}m1$	4.210	4.210	7.731	90.00	90.00	120.00

BaF_2	$Fm\bar{3}m$	4.442	4.442	4.442	60.00	60.00	60.00
$BaCl_2$	$Fm\bar{3}m$	5.242	5.242	5.242	60.00	60.00	60.00
BaI_2	$Pnma$	5.418	10.848	18.051	90.16	90.00	90.00
KBr	$Fm\bar{3}m$	4.749	4.749	4.749	60.00	60.00	60.00
SiC	$P6_3mc$	3.093	3.093	15.178	90.00	90.00	120.00
SiO_2 (quartz)	$I\bar{4}2d$	5.036	5.036	7.371	90.00	90.00	90.00
TiO_2	$\frac{C2}{c}$	3.803	3.803	9.734	90.00	90.00	90.00
ZnS (sphalerite)	$F\bar{4}3m$	5.431	5.431	5.431	90.00	90.00	90.00

Supplementary Table 5. Bader charges and degrees of ionicity in some molecules determined from experimental dipole moments, from Bader analysis, and calculated using Pauling's and our formulae.

	Bader Charges		Ionic Character / %			
System	Cation	Anion	From dipole moment	From Bader analysis	From Pauling's formula	From our formula
HF	0.72	-0.72	41.33	71.62	54.71	45.90
HCl	0.38	-0.38	17.64	37.67	20.58	13.16
HBr	0.42	-0.42	12.18	42.06	13.45	10.60
HI	0.44	-0.44	5.80	43.56	5.15	1.69
LiF	0.70	-0.70	83.66	69.82	89.46	89.28
LiCl	0.71	-0.71	72.99	71.19	69.52	69.25
LiBr	0.79	-0.79	69.34	79.28	62.47	66.45
LiI	0.84	-0.84	54.68	84.14	50.62	50.70
NaF	0.63	-0.63	87.82	62.87	90.23	89.79
KCl	0.88	-0.88	79.94	88.25	74.56	74.42
KBr	0.90	-0.90	78.26	89.98	68.17	71.91
KI	0.90	-0.90	75.49	90.14	57.10	57.31
CsF	0.73	-0.73	69.87	72.82	92.15	93.59

CsCl	0.80	-0.80	74.51	80.23	75.44	79.00
CsI	0.85	-0.85	76.00	85.04	58.28	63.53
ICl	0.03	-0.03	11.12	3.16	6.06	5.82
IBr	0.10	-0.10	6.22	9.82	2.22	4.08

Supplementary Table 6. Bader charges and degrees of ionicity of some crystals determined using Bader analysis and calculated using Pauling's and our formulae.

	Bader Charges		Ionic Character / %		
System	Cation	Anion	From Bader analysis	From Pauling's formula	From our formula
AlN	2.37	-2.37	78.93	40.02	51.38
Al_2O_3	2.48	-1.65	82.62	56.71	65.30
BaF_2	1.72	-0.86	86.07	90.81	92.67
$BaCl_2$	1.62	-0.81	80.38	72.42	76.78
BaI_2	1.55	-0.78	77.71	54.31	60.48
BaO	1.56	-1.56	78.18	80.32	87.32
BeF_2	1.76	-0.88	87.89	76.59	81.07
$BeCl_2$	1.67	-0.83	83.29	46.85	54.05
BeI_2	1.54	-0.77	76.79	25.70	33.34
BeO	1.69	-1.69	84.45	58.28	70.86
Bi_2Te_3	0.70	-0.47	23.49	0.16	12.63
CsF	0.88	-0.88	88.13	92.15	93.59
CsCl	0.84	-0.84	84.29	75.44	79.00

CsI	0.81	-0.81	80.69	58.28	63.53
KBr	0.83	-0.83	82.90	68.17	71.91
LiH	0.83	-0.83	83.23	31.07	39.63
LiF	0.87	-0.87	86.82	89.46	89.28
LiCl	0.89	-0.89	88.51	69.52	69.25
LiI	0.88	-0.88	88.28	50.62	50.70
MgF_2	1.67	-0.84	83.53	83.17	82.24
$MgCl_2$	1.66	-0.83	82.84	57.50	56.02
MgI_2	1.58	-0.79	79.19	36.59	35.43
MgO	1.64	-1.64	82.19	67.83	72.42
NaF	0.82	-0.82	82.43	90.23	89.79
NaCl	0.83	-0.83	82.91	71.15	70.33
NaI	0.82	-0.82	82.40	52.68	52.05
PbH	0.37	-0.37	37.40	2.69	11.09
RhH	0.10	-0.10	9.61	0.16	9.64
SiC	2.64	-2.64	66.10	10.02	7.00
SiO_2 (quartz)	3.22	-1.61	80.45	44.73	45.90

TiO_2	1.98	-0.99	49.54	59.44	79.84
ZnS (sphalerite)	0.88	-0.88	43.97	19.44	60.48

Supplementary Figure 7 Correlation between different electronegativities with work function[7] of pure elements. *Legend*: metals, empty circles; non-metals, full squares.

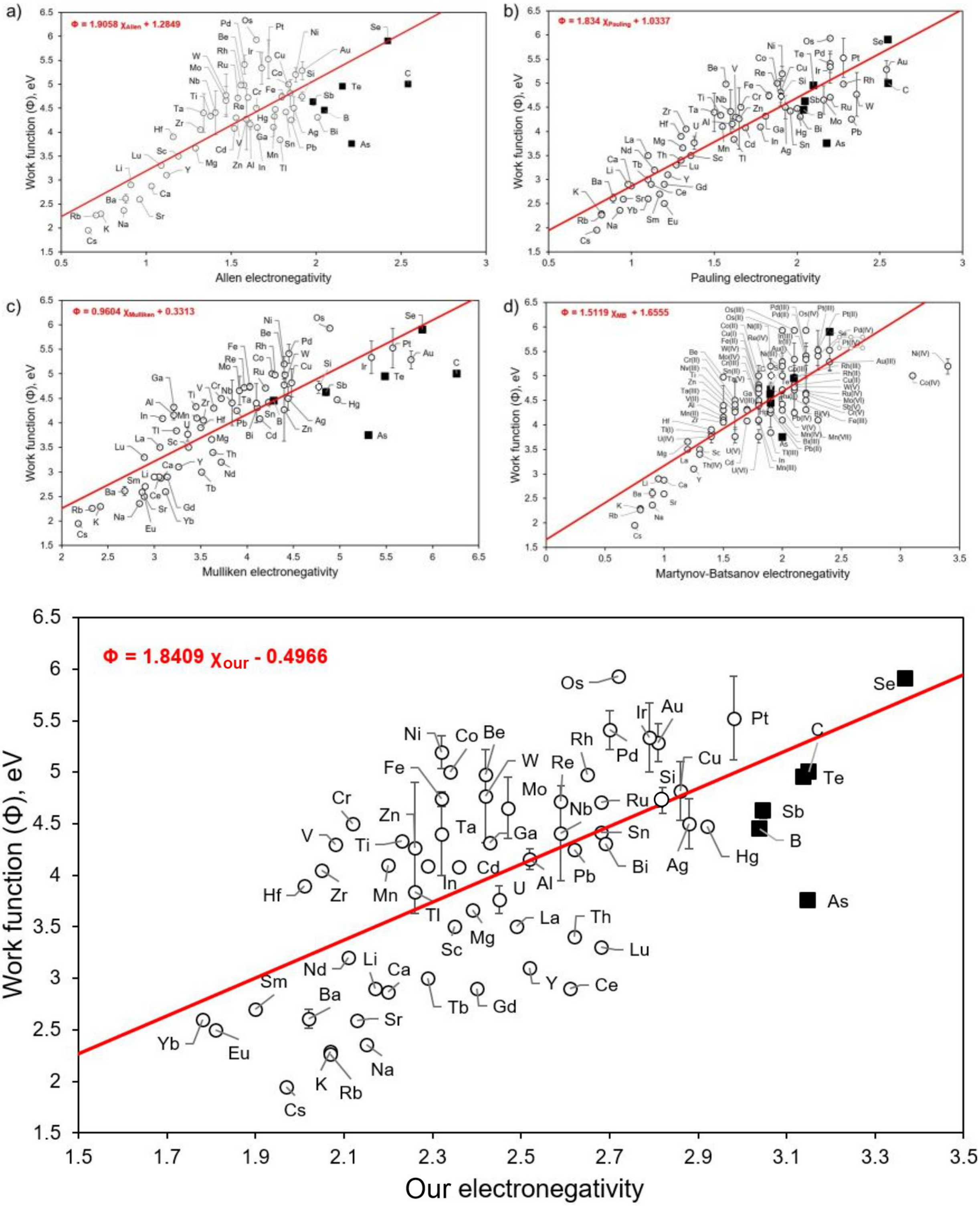

(e)
