# Supplementary material for: Thermochemical electronegativities of the elements
Source: Nat Commun. 2021 Apr 7;12:2087. doi: 10.1038/s41467-021-22429-0 (PMC8027013; doi:10.1038/s41467-021-22429-0)
Supplement: Supplementary file 2 — Description of Additional Supplementary Files [file 41467_2021_22429_MOESM2_ESM.docx]

File Name: Supplementary Data 1

Description: Electronegativities of the elements from different scales. For Martinov-Batsanov scale there are different electronegativity values for the different oxidation states (reported in brackets) of each element.
